# Cytokine and Growth Factor Delivery from Implanted Platelet-Rich Fibrin Enhances Rabbit Achilles Tendon Healing

**DOI:** 10.3390/ijms21093221

**Published:** 2020-05-02

**Authors:** Chin-Chean Wong, Yu-Min Huang, Chih-Hwa Chen, Feng-Huei Lin, Yi-Yen Yeh, Meng-Yi Bai

**Affiliations:** 1 Department of Orthopedics, Shuang Ho Hospital, Taipei Medical University, New Taipei City 23561, Taiwan; b8701153@tmu.edu.tw (C.-C.W.); yellowcorn0326@yahoo.com.hk (Y.-M.H.); afachen@doctor.com (C.-H.C.); 2 Department of Orthopedics, School of Medicine, College of Medicine, Taipei Medical University, Taipei 11031, Taiwan; 3 Research Center of Biomedical Devices, Taipei Medical University, Taipei 11031, Taiwan; 4 International Ph.D. Program for Cell Therapy and Regenerative Medicine, College of Medicine, Taipei Medical University, Taipei 11031, Taiwan; 5 Department of Biomedical Engineering National Taiwan University, Taipei 10617, Taiwan; double@ntu.edu.tw; 6 School of Biomedical Engineering, College of Biomedical Engineering, Taipei Medical University, Taipei 11031, Taiwan; yan0228@tmu.edu.tw; 7 School of Medicine, College of Medicine, Taipei Medical University, Taipei 11031, Taiwan; 8 Institute of Biomedical Engineering & Nanomedicine, National Health Research Institutes, Miaoli County 35053, Taiwan; 9 Graduate Institute of Biomedical Engineering, National Taiwan University of Science and Technology, Taipei 10607, Taiwan

**Keywords:** implantation, cytokine-delivery, platelet-rich fibrin, Achilles tendon, healing

## Abstract

Tendons are hypocellular and hypovascular tissues, and thus, their natural healing capacity is low. In this study, we sought to evaluate the efficacy of platelet-rich fibrin (PRF) to serve as a bioactive scaffold in promoting the healing of rabbit Achilles tendon injury. For in vitro study, the essence portion of PRF was determined through bioluminescent assay. Furthermore, we analyzed the time-sequential cytokines-release kinetics of PRF and evaluated their effects on tenocytes proliferation and tenogenic gene expressions. In animal study, the rabbit Achilles tendon defect was left untreated or implanted with normal/heat-denatured PRF scaffolds. Six weeks postoperatively, the specimens were evaluated through sonographic imaging and histological analysis. The results revealed significantly more activated platelets on bottom half of the PRF scaffold. Cytokine concentrations released from PRF could be detected from the first hour to six days. For the in vitro study, PRF enhanced cell viability and collagen I, collagen III, tenomodulin, and tenascin gene expression compared to the standard culture medium. For in vivo study, sonographic images revealed significantly better tendon healing in the PRF group in terms of tissue echogenicity and homogeneity. The histological analysis showed that the healing tissues in the PRF group had more organized collagen fiber, less vascularity, and minimal cartilage formation. In conclusion, bioactive PRF promotes in vitro tenocytes viability and tenogenic phenotypic differentiation. Administration of a PRF scaffold at the tendon defect promotes tissue healing as evidenced by imaging and histological outcomes.

## 1. Introduction

The Achilles tendon is the largest and strongest tendon in the human body and helps to transmit forces from the calf muscles to the calcaneus during motion [1]. Achilles tendon injuries are common not only in athletes but also in non-athlete adults. The treatment of acute Achilles tendon injuries can be categorized into operative and non-operative. Regardless of the treatment method, the primary goal of management of acute Achilles tendon injuries is to ensure a rapid return to full function. Nonetheless, tendon healing usually results in the formation of fibrovascular scar or granulation tissue. This discrepancy in the microstructure and biomechanical properties of scar tissues often makes the tissue more susceptible to re-injury or even re-rupture [2,3,4,5].

Currently, therapies utilizing biological augmentation techniques are being investigated for potential benefits in tendon healing. Certain growth factors could act directly on target cells present in the injured site to facilitate the healing process. These include insulin-like growth factor-1 (IGF-1), basic fibroblast growth factor (bFGF), platelet-derived growth factor (PDGF), and transforming growth factor-β (TGF-β) [2,3,4,5,6,7,8]. However, their clinical use is still limited because of their high costs, short preservation period, and limited clinical availability. Platelet-rich plasma (PRP) is a derivative of the patient’s own blood that contains a supraphysiological concentration of platelets [9]. Platelets contribute to injury healing by releasing an ordered sequence of growth factors, cytokines, and an array of bioactive proteins in soluble and membrane-bound forms over the lifespan of the platelets. Many studies have revealed the positive role of PRP in promoting tendon healing [10,11]. However, the major drawback of using PRP clinically is that when PRP is applied via liquid injection, the required mechanical environment of tendinous regeneration is difficult to maintain. Moreover, PRP implanted with either synthetic or biological scaffolds may incur unexpected risk of adverse immune reactions [12].

Platelet-rich fibrin (PRF) is a second-generation platelet concentrate derived from autologous blood obtained immediately after centrifugation but without anticoagulant supplement [13,14]. This self-scaffolding PRF gel is a three-dimensional (3D) fibrin biomaterial containing a high concentration of growth factors, including TGF-β, BMP-2, IGF-1, and PDGF [15,16]. Unlike PRP, which needs additional scaffolding for tissue in situ transplantation, PRF is a strictly autogenous fibrin-based biomaterial that enables the local and progressive delivery of growth factors, which can be used to enhance tissue healing and regeneration [17]. 

In previous studies, the effectiveness of a PRF scaffold in promoting meniscal and cartilaginous repairs by enhancing cell proliferation, migration, differentiation, and matrix synthesis has been demonstrated [16,18,19]. To our knowledge, limited studies have combined preliminary in vitro with an in vivo study evaluating the efficacy of PRF in promoting Achilles tendon healing, and the results are inconsistent [20,21,22,23]. In this pre-clinical trial, we hypothesized that PRF could serve as a “cytokine delivery vehicle” that would be beneficial for tendinous healing. For the in vitro portion, we determine the essence portion of PRF as well as its time-sequential cytokine-release kinetics. Moreover, PRF effects on tenocytes proliferation, tenogenic gene, and protein expression were studied. Finally, the effects of the PRF scaffold in augmenting Achilles tendon healing in a rabbit model were evaluated through sonographic and histological assessments. 

## 2. Results

### 2.1. In Vitro

#### 2.1.1. Quantification of ATP Contents Released from Activated Platelets 

**Figure 1:**
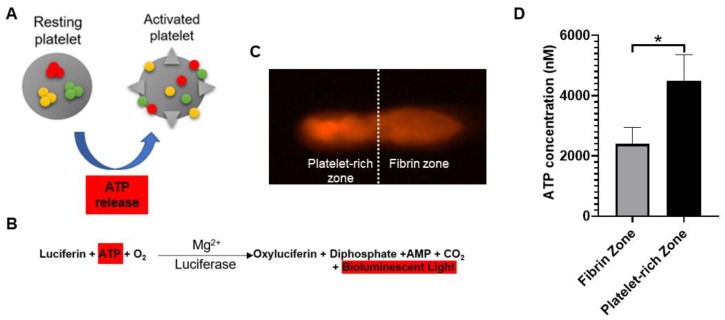
(**A**) Adenosine triphosphate (ATP) is released from dense granules during platelet activation. (**B**) Detection of activated platelets on platelet-rich fibrin (PRF) through bioluminescent assay. (**C**) PRF sample was observed under a fluorescence optical microscope. Based on bioluminescent intensity, the PRF could be equally divided into fibrin zone and platelet-rich zone. (**D**) The image brightness of bioluminescence was substituted into the ATP standard curve to determine the concentration of platelet-released ATP. The bars show the mean ± SD (*n* = 5) of each group. * *p* < 0.05.

When platelets are activated by thrombotic stimuli, their contained ATP is secreted extracellularly (Figure 1A) [24]. Therefore, the bioactivity of PRF was determined through determining local adenosine triphosphate (ATP) released from activated platelets. Figure 1B showed the reaction equation of firefly luciferase assay. The assay is based on luciferase’s requirement for ATP in producing light (emission maximum ~560 nm at pH 7.8). Figure 1C showed the acquired bioluminescence imaging of PRF sample observed under fluorescence optical microscope. The bioluminescence light can be detected across the entire PRF gel, and the image brightness was recorded and quantitated. According to the reaction equation, the light production by cell-surface-attached luciferase is continuous and linearly related to ATP concentration. Figure 1D showed the ATP concentrations of PRF samples derived from five different rabbits.

#### 2.1.2. Time-Sequential Cytokine Release 

**Figure 2:**
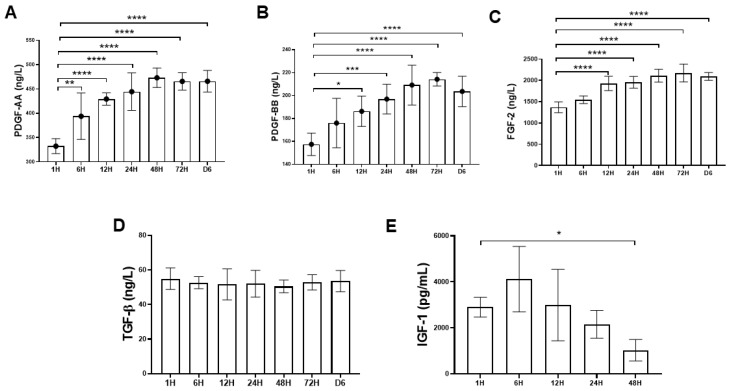
Time-sequential release profile of platelet-derived growth factor-AA (PDGF-AA), platelet-derived growth factor-BB (PDGF-BB), fibroblast growth factor-2 (FGF-2), transforming growth factor-β (TGF-β), and insulin-like growth factor-1 (IGF-1) from first hour to six days. The PDGF-AA, PDGF-BB, FGF-2, and TGF-β1 releases were sustained over six days. (**A**–**C**) The PDGF-AA, PDGF-BB, and FGF-2 releases were rapidly induced, and a maximum concentration was detected at 48 h and 72 h. (**D**) The TGF-β1 release was constant and sustained over six-day period. (**E**) The IGF-1 release decreased over time and could not be detected after 48 h. The bars show the mean ± SD fold change (*n* = 6) of each group. * *p* < 0.05; ** *p*< 0.01; *** *p* < 0.001; **** *p* < 0.0001.

Figure 2A–E showed the time-sequential release pattern of five different types of cytokine starting from 1 h until day 6. The results demonstrated that platelet-derived growth factor-AA (PDGF-AA), platelet-derived growth factor-BB (PDGF- BB) and fibroblast growth factor-2 (FGF-2) content released from PRF increased significantly over the time course of the study. For PDGF-AA and PDGF-BB, the cytokine concentrations peaked at 48 h and 72 h, respectively, with mean values of 473.12 ± 19.8 ng/L and 214.26 ± 5.82 ng/L (Figure 2A,B). For FGF-2, the cytokine concentration gradually increased and reached top at 72 h, with mean values of 2168.14 ± 83.5 ng/L (Figure 2C). In contrast, the transforming growth factor-β (TGF-β) concentration remained stable without much fluctuation with mean value of 52.6 ± 1.42 ng/L (Figure 2D). The level of insulin-like growth factor-1 (IGF-1) decreased after 6 h and could not be detected after 48 h (Figure 2E). 

#### 2.1.3. Dose-Dependent Effects of PRF on Tenocytes Viability and Proliferation 

**Figure 3:**
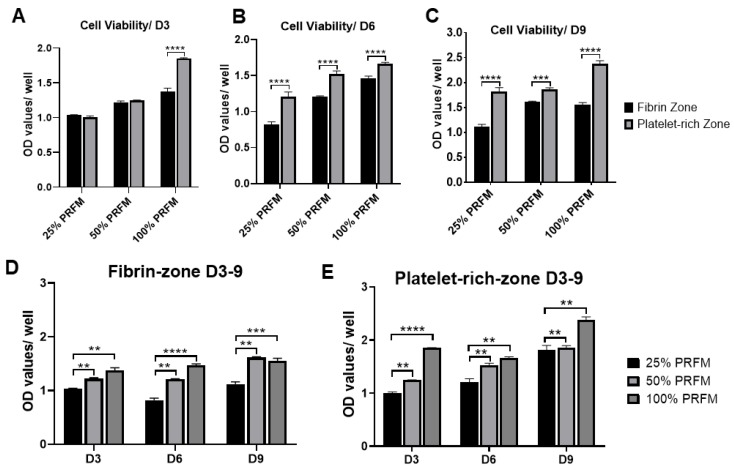
Differential effects of fibrin zone- and platelet-rich zone-platelet-rich fibrin conditioned medium (PRFM) on viability of tenocytes. (**A**–**C**) The MTT (3-(4,5-dimethylthiazol-2-yl)-2,5-diphenyltetrazolium bromide) assay was used to assess the viability of tenocytes under fibrin zone- and platelet-rich zone PRFM treatments at three different time points. On day 6 and day 9, platelet-rich zone PRFM was more potent than fibrin zone PRFM in promoting cell growth, regardless of PRFM concentration. (**D**,**E**) Dose-dependent effects of fibrin zone- and platelet-rich zone PRFM on viability of tenocytes during a nine-day culture. The bars show the mean ± SD fold change (*n* = 4) of each group. D3, day3, D6, day 6, D9, day 9. * *p* < 0.05; ** *p* < 0.01; *** *p* < 0.001; *** *p* < 0.0001.

The MTT (3-(4,5-dimethylthiazol-2-yl)-2,5-diphenyltetrazolium bromide) assay was used to evaluate the differential effects of two types of PRF-conditioned medium (PRFM), known as the fibrin zone PRFM and platelet-rich zone PRFM on tenocytes viability during nine-day cultivation. Figure 3A–C shows that significantly higher cell viability and proliferation were noted in tenocytes treated with platelet-rich zone PRFM compared to fibrin zone-PRFM, particularly on day 6 and day 9. On day 6, the 25% (1.21 + 0.06), 50% (1.52 ± 0.05), and 100% platelet-rich zone PRFM (1.66 ± 0.02) has a 1.2- to 1.5-fold increase in cell numbers compared to fibrin-zone-PRFM. On day 9, the potent effects of platelet-rich zone PRFM on cell viability remained comparable to its counterpart fibrin-zone PRFM (*p* < 0.001). The data clearly displayed the differential effects of two different PRFMs on cell viability, suggesting that platelet-rich zone-PRF may contain a higher amount of cytokines responsible for increased cell growth. Figure 3D,E confirms that increasing concentrations of PRFM did increase the cell viability of cultured tenocytes in a dose-dependent manner, both in fibrin zone PRFM and platelet-rich zone PRFM groups.

#### 2.1.4. PRF Promotes Tenogenic Gene Expression 

**Figure 4:**
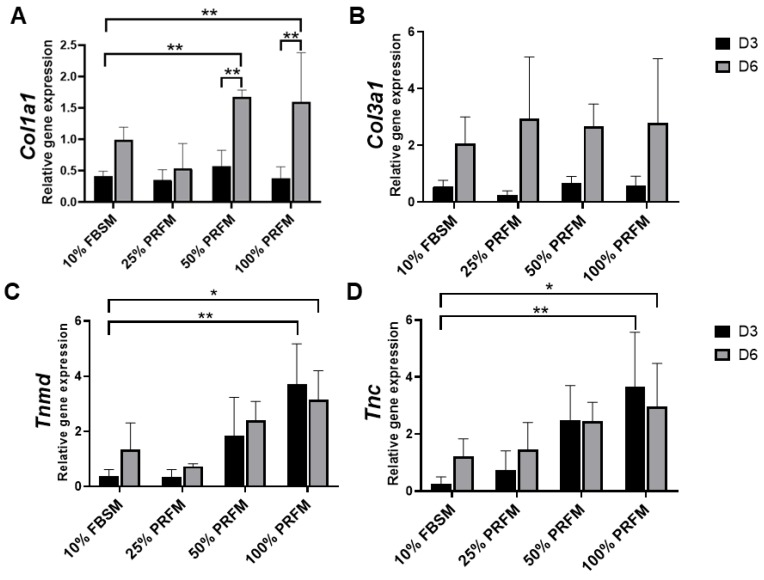
Quantitative expression profiles of (**A**) Collagen I (*Col1a1*), (**B**) Collagen III (*Col3a1*), (**C**) Tenomodulin (*Tnmd*), and (**D**) Tenascin (*Tnc*) of cultured tenocytes on day 3 and day 6. The asterisks indicate significant differences in the gene expression among all four groups: Dulbecco’s Modified Eagle’s Medium F-12 containing 10% fetal bovine serum (10% FBSM), and 25%, 50%, and 100% platelet-rich fibrin medium (PRFM) prepared from platelet-rich zone PRF. The bars show the mean ± SD fold change (*n* = 4) of each group. D3, day3, D6, day 6. * *p*< 0.05; ** *p*< 0.01.

In this experiment, the PRFMs prepared from platelet-rich zone was used. In addition, the effects of different concentration of PRFM was compared with standard culture medium (DMEM/F-12 containing 10% FBS (10% FBSM)). The type I collagen (*Col1a1*) expression was upregulated from day 3 to day 6 in 50% and 100% PRFM groups (Figure 4A). No significant difference in type III collagen (*Col3a1*) expression was detected between 10% FBSM and PRFM groups (Figure 4B). As shown in Figure 4C,D, treatment of tenocytes with 100% PRFM significantly upregulated Tenomodulin (*Tnmd*) and Tenascin (*Tnc*) expressions. Compared with 10% FBSM, the 100% PRFM-treated tenocytes demonstrated 10-fold and 2.3-fold increases in *Tnmd* expression on days 3 and 6, respectively. On the other hand, the 50% and 100% PRFM group has 13.5-fold and 2.5-fold increase in *Tnc* expression compared with 10% FBSM after six-day cultivation. The results indicated that expression of tenogenic markers increased in the PRFM culture was correlated with PRFM concentration. 

#### 2.1.5. Sonographic Findings

**Figure 5:**
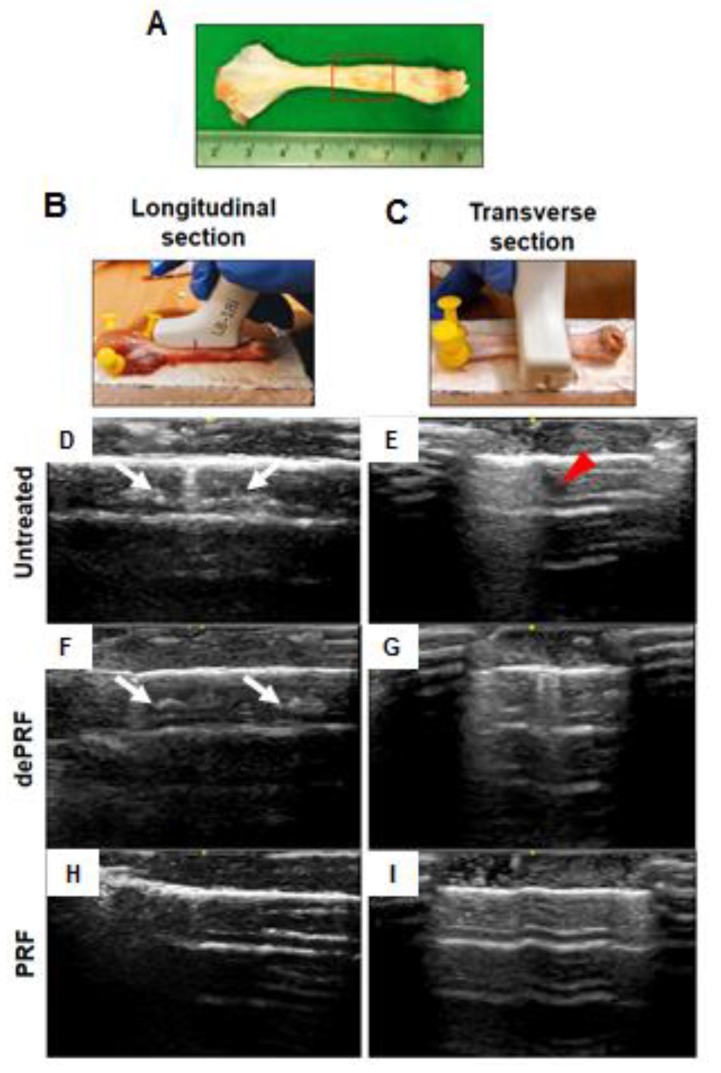
Sonographic evaluation of the healing process of rabbit Achilles tendon after platelet-rich fibrin (PRF) implantation. (**A**) Gross view of tendon specimens. The red rectangular frame highlighted the repaired zone. (**B**,**C**) The tendon specimens were evaluated longitudinally (**B**) and transversely (**C**). Tendon sonographic images in the untreated control (**D**,**E**), the heat-denatured PRF group (dePRF) (**F**,**G**), and the PRF group six weeks after treatment (**H**,**I**). The white arrows indicate intratendinous hyperechoic areas suggesting scar tissue formation. The red arrowhead indicates intratendinous fluid accumulation.

The sonographic findings were evaluated based on the intratendinous morphology of the repaired tendon (Figure 5A–C). In the untreated group and heat-denatured PRF (dePRF) group, the longitudinal and transverse views showed that the defect region demonstrating inhomogeneous echo structure. Some anechoic areas were detected as discontinuous, fibrillary echo texture resulting from incomplete healing of tendon defects (Figure 5D–G). Moreover, some intratendinous hyperechoic areas which are suggestive of scar tissue formation or calcifications were also noted (Figure 5D–G). In the PRF group, the defect region demonstrating continuous fibrillary appearance with tendon fiber are well aligned along the long axis of the native tendon (Figure 5H,I). 

#### 2.1.6. Histological Analysis of Tendon Healing 

**Figure 6:**
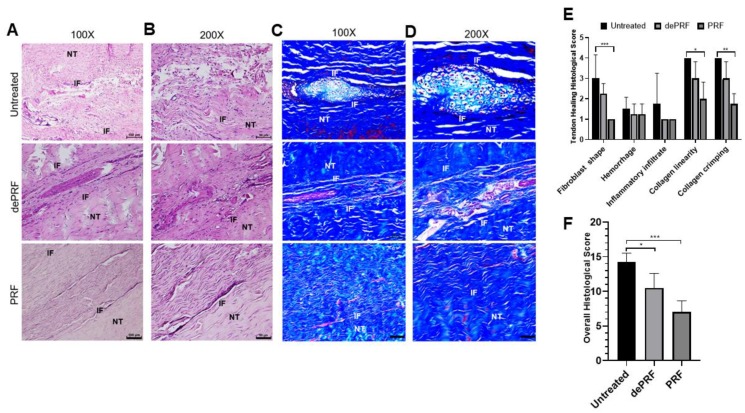
Histological evaluation of tendon healing after post-operative 6 weeks by hematoxylin and eosin (H&E) and Masson Trichrome (MT) staining. (**A**,**B**) The H&E staining of repaired tissue of untreated control (upper panel), heat-denatured PRF (dePRF) (middle panel), and PRF groups (lower panel). (**C**,**D**) Characterization of matrix components formed at the repaired zone by MT staining in the untreated control (upper panel), heat-denatured PRF (dePRF) (middle panel), and PRF groups (lower panel). (**E**,**F**) Histological scores of repaired tissue in three groups. NT, normal tendon; IF, interface. Scale bar: A, C: 100 μm; B, D: 50 μm. The bars show the mean ± SD (*n* = 3) of each group. * *p*< 0.05; ** *p*< 0.01; *** *p* < 0.001.

Figure 6A–D shows the histological images of the repaired tissues at three groups stained by hematoxylin and eosin (H&E) and Masson Trichrome (MT) at different magnification powers. In the untreated group, a cluster of round-shaped chondrocyte-like cells was found at the repaired zone. Moreover, the fibers were loosely composed without significant order (upper panel, Figure 6A–D). In dePRF group, the repaired zone was filled with heterogeneous tissues formed by fragmented collagen bundles. The tissues consist of cells in different shapes and sizes (middle panel, Figure 6A–D). The cartilage-like tissues and granulation tissues respective to untreated group and dePRF group were scarcely stained by MT. In the PRF group, most of the cells at the repaired zone were elongated in shape, resembling normal tenocytes. The cellularity is remarkably higher, and the collagen fibers were wavy and formed a crimping pattern (lower panel, Figure 6A–D). Moreover, the extracellular matrix composition at the repaired zone in the PRF group was strongly stained by MT, indicating dense collagen formation. 

Quantitatively, the individual evaluation of five parameters revealed that in PRF group, the cells at the repaired zone were more fibroblast like cells resided in organized collagen fibers (Figure 6E). The overall histological score of dePRF (10.5 ± 2.6) and the PRF group (7 ± 1.6) were better than the untreated group (14.25 ± 1.26, *p* < 0.05) (Figure 6F). No significant difference in vascular hyperplasia and inflammatory infiltration were noted among the three groups. 

## 3. Discussion

In the past few decades, platelet concentrate has emerged as an adjunct therapeutic for musculoskeletal injury. The rationale for its use is largely dependent on its functional components, which include a variety of growth factors, coagulation factors, adhesion molecules, cytokines, and chemokines [14,25,26,27,28]. Upon activation, the platelets can release these anabolic growth factors at concentrations significantly higher than the baseline blood levels [27].

The aims of this present study are to demonstrate the utility and capacity of PRF to serve as a “cytokine-delivery vehicle” in promoting Achilles tendon healing. In our previous studies, PRF demonstrated pronounced effects on cellular migration, viability, and differentiation both in vitro and in vivo, which may translate into a viable strategy in promoting tendinous healing. Nonetheless, the cytokine-release kinetics of PRF and its influence on tendinous healing remained largely unknown. Based on our results, the essence portion of PRF was successfully defined and its bioactivity on cultured tenocytes were revealed. When implanted in vivo, PRF can improve functional tendinous healing evidenced by imaging and histological results. From a clinical standpoint, our data demonstrate a new and effective way to improve tendinous healing.

Platelet-rich fibrin (PRF) is a second-generation platelet concentrate produced from autologous blood obtained immediately after single-spin centrifugation [14]. Rapid activation of the coagulation cascade and synthesis of thrombin take place when the platelets come into contact with the glass particles of the test tube. The ATP is co-packaged in platelet–dense granules with serotonin, Ca^2+^, and ADP and are secreted when the platelets are activated [24,29]. In the current study, the distribution of activated platelets on the PRF scaffold was identified by detecting the intensity of emitted bioluminescence light transients produced through the reaction of luciferin and activated platelet–released ATP. Geographically, we found that there were two distinct zones in the PRF scaffold in terms of bioluminescence intensity, known as the fibrin zone located at the upper half and the platelet-rich zone at the bottom half. Regarding PRF microstructure, some authors have revealed the inconsistency of scaffold compactness and porosities [26,30]. Therefore, we assume that the accumulation of activated platelets at the bottom half of PRF scaffold are attributed to the tighter compactness and smaller porosities of fibrin meshwork followed centrifugation. From functional perspective, the bottom-half platelet-rich zone was thus defined as the “PRF essence” region due to the relatively higher number of platelets. Despite the variations in PRF cytokine from batch to batch, the PRF essence consistently contains highly concentrated platelets and cytokines. When applied in vivo, we believe that the features of PRF essence may compensate for the individual discrepancy to facilitate tendinous healing.

Since platelets aggregate along the fibrin fibers during clotting, the resultant three-dimensional (3D) scaffold could act as a reservoir of growth factors. Moreover, the equilateral junctions of the PRF 3D matrix allow the establishment of a fine and flexible fibrin network that enable cytokines enmeshment [14]. Despite the increase in the clinical use of the platelet concentrates such as PRP for local tissue healing and regeneration, little is known about the biomolecule characteristic of these therapeutics in terms of cytokine-release kinetics. In this study, growth factors such as PDGF (AA and BB isoforms), FGF-2, TGF-β1, and IGF-1 were chosen for analysis because there are basic cytokines identified in platelets that play crucial role in cell proliferation, differentiation, and chemotaxis [5]. In this study, the time-sequential cytokine-release kinetics was revealed by determining the cytokine concentrations of PRF-immersed supernatant at different time points. It was demonstrated that most growth factors could be detected at the first hour. Interestingly, an increasing trend of PDGF-AA and BB, and FGF-2 release was detected even after six days. The results of this study were consistent with previous reports, suggesting that the PRF tightly packed fibrin fibers can exhibit a locking effect on the cytokines [30,31]. The results of this study also lend support to our hypothesis that PRF could serve as a cytokine-delivery vehicle that would sustainably deliver bioactive molecules in promoting tendinous healing.

The functional healing of tendinous injury relied on the formation of tendinous tissue composed of sufficient cells with tenogenic potential. In this study, we found that PRF-conditioned medium (PRFM) could stimulate tenocytes proliferation and tenogenic matrix production. Based on the cytokine-release kinetic analysis, we found that the increased anabolic activities of tenocytes may be attributed to the cytokines originating from PRF. PDGF is a powerful mitogen for fibroblast which could increase cell density and proliferation [32]. FGF could increase production of extracellular matrix while applied in vivo [33]. When IGF-1 and TGF-β were delivered to the repair site of supraspinatus tendon-to-bone insertions of rats, increased cell proliferation, vascularity, and the production of fibrous repair tissue could be observed [34]. Our results showed that the number of viable tenocytes treated with proportionally increased concentrations of PRFM (25%, 50%, and 100%) increased in a dose-dependent manner during the nine-day culture (Figure 3). On the other hand, the expression of tendon-related genes such as *Tnmd* and *Tnc* were significantly upregulated after treatment with high-concentration PRFM. *Tnmd* is a gene that is highly expressed in tendons and is required for tenocytes proliferation and tendon maturation [35,36,37]. *Tnc* is a glycoprotein that plays an important role for tenocytes adaptation to compression [38]. Moreover, PRFM has comparable effects as 10% FBSM on *Col1a1* and *Col3a1* gene expressions. Both types of collagen are important molecules in the tendon extracellular matrix and are produced in high quantity at the early phase of tendinous healing [2,3,39]. Collectively, the in vitro results indicated that PRF exerts anabolic effects on tenocytes proliferation and tenogenic differentiation, which would benefit for in vivo tendinous healing.

PRF is a biomaterial yielded by a natural polymerization process during centrifugation [14]. Its natural fibrin architecture is beneficial for the containment and slow release of growth factors over time [13,30]. When implanted in vivo, the PRF scaffold could serve as a delivery platform for growth factors and provides an architecture for the attachment, proliferation, and migration of cells at the target site. The in vivo results demonstrated superior tendinous healing in the PRF-implanted group compared to control and the dePRF-implanted group, evidenced by sonographic and histological findings. For sonographic analysis, the intratendinous hyperechoic areas found in the control and dePRF groups may be ascribed to scar tissues comprised of different types of cells and an unorganized matrix. In contrast, the intratendinous morphology of the repaired tendon in PRF are mostly isoechoic. Moreover, the repaired tissue displayed a fibrillary appearance. These imaging findings indicate that the collagen fibers of the repaired tissue are cross linked and align along the long axis of the tendon, suggesting that the remodeling phase was underway.

In general, tendon healing progresses through an inflammatory phase (days), followed by a reparative phase (weeks), and ended with a remodeling phase (months) [4,39]. Our histological results showed that PRF implantation leads to the initiation of a tendon reparative process characterized by increased tenocytes proliferation and differentiation. Despite the relatively high number of proliferative tenocytes found at the healing zone, most of the cells are spindly and elongated in shape. In addition, the collagen fibers were continuous, wavy, and aligned longitudinally in one direction. On the other hand, in control and dePRF rabbits, most of the tenocytes are oval/round in shape, resembling chondrocytes-like cells with basophilic lacanue. It was reported that the presence of cartilage is a reliable indicator of an inferior repair process and poorer biomechanics [40]. Moreover, the fiber arrangements at the healing zone was loosed, disorganized, and fragmented without an identifiable pattern. Taken together, it was reasonably to propose that PRF could improve the reparative process by upregulating the tenocytes growth and collagen production. However, the histological findings also suggest that the remodeling process of tendinous injury is yet to be completed in terms of increased cell number and immature matrix synthesis.

Our data showed the potential of PRF in promoting Achilles tendon healing in a preclinical trial with an animal model. However, our study has several limitations. First, this preliminary study was conducted to evaluate the biological role of PRF in tendinous healing process at six weeks postoperatively. However, it might be more thorough to assess the surgical repair outcomes over different time points. The results also indicate that a longer study period may be needed to evaluate the complete course of tendinous healing.

## 4. Materials and Methods

### 4.1. Study Design and Ethics Statement

All procedures of tenocytes isolation and surgery on experimental animals were carried out according to the guide for the Care and Use of Laboratory Animals and was approved by the Institutional Animal Care and Use Committee (IACUC approval: LAC-2016-0247, 10 Feb 2017).

### 4.2. In Vitro

#### 4.2.1. Preparation of Platelet-Rich Fibrin (PRF)

The PRF gel was prepared using the technique described by Choukroun et al. [13]. All PRF gel used for in vitro and in vivo testing were harvested from donor rabbits. Briefly, 8 mL of venous blood was collected in a sterile tube without anticoagulant supplement through venipuncture of the rabbit’s marginal ear vein. The blood collection tubes were then centrifuged at 400 g for 10 min in a DSC-200A-2 tabletop centrifuge (Digisystem, Laboratory Instruments Inc., New Taipei City, Taiwan). The acquired PRF gel was located between the red blood cells and the acellular plasma. Under sterile condition, the PRF gel was retrieved from the tube and then transferred to a sterile 15 mL centrifuge tube and stored at −80 °C before further analysis.

#### 4.2.2. Detection of local adenosine triphosphate release from activated platelets

The detection method was first described by Beigi et al. [24]. In this study, a bioluminescent firefly luciferase assay (A22066, Thermo Fisher Scientific Inc., Waltham, Ma, USA) was used to determine the released ATP content of PRF gel following the manufacturer’s instructions. Based on the reaction principle, a greater bioluminescence indicates more activated platelets entrapped within the PRF gel. First, this kit supplies a 20× concentrate of reaction buffer dithiothreitol (DTT) reaction agent for use in the reaction and an ATP solution for preparing standard curves. Thus, a standard curve of ATP concentration versus luminescence was established to quantitatively determine the concentration of ATP secreted by activated platelets. For quantitative analysis, the PRF gel was immersed with reaction solution for 15 min and immediately subjected for image acquisition by a macro imaging system (LT-9MACIMSYSPLUSC, Lightools Research, South Encinitas, CA, USA). The yield bioluminescence intensity was then substituted into the ATP standard curve to determine the concentration of ATP.

#### 4.2.3. Cytokine Quantitation and Time-Sequential Release Kinetics

In order to determine the amount of released cytokine from platelets, each PRF sample (*n* = 6) was immersed in 5 mL of phosphate buffered saline (PBS) and placed into a shaking incubator at 4 °C to allow for cytokine release into PBS, starting from 1 h, 6 h, 12 h, 24 h, 48 h, 72 h to six days. At each time point, the aliquots were collected, frozen, and replaced with 5 mL of additional PBS. At designated time point, the PDGF-AA (range: 5–1000 pg/mL), PDGF-BB (range: 2–600 pg/mL), FGF-2 (range: 20–6000 pg/mL), TGF-β (range: 15–240 pg/mL), and IGF-1 (range: 0.156–10 ng/mL) concentrations were quantitated using commercially available bead-based sandwich immune-assay kits (PDGF AA & BB, FGF-2: Bioassay Technology Laboratory, Shanghai, China; TGF-β: Abbkine, CA, USA; IGF-1: Cloud-clone, Katy, TA, USA).

#### 4.2.4. Preparation of PRF-Conditioned Medium

Two types of PRF-conditioned medium (PRFM) were prepared, known as the fibrin zone PRFM and platelet-rich zone PRFM. First, the thawed PRF gel was cut perpendicularly followed the long axis and equally divided into fibrin zone PRF and platelet-rich zone PRF. Then, the divided PRF was soaked independently in 10 mL of serum-free Dulbecco’s Modified Eagle’s Medium without fetal bovine serum (FBS) supplement (DMEM/F-12 medium) (Gibco, Paisley, UK) in a centrifuge tube, defined as 100% PRFM. The tubes were then put into a tube rotator at 4 °C for 24 h. The conditioned medium acquired either from fibrin zone-PRF or platelet-rich zone-PRF was then filtered and diluted proportionally to 50% and 25%, known as 50% PRFM and 25% PRFM, respectively.

#### 4.2.5. Isolation and Culture of Rabbit Tenocytes

Using aseptic technique, the rabbit heel cord was approached through a longitudinal incision. The Achilles tendons were detached and harvested from its bony attachments and was washed in the growth medium containing DMEM F-12 medium supplemented with 10% FBS, 100 U/mL penicillin, and 100 mg/mL streptomycin. Tendon tissue was minced into small fragments and digested with collagenase (100 UI/mL Gibco, Paisley, UK) for 2 h at 37 °C. The digestion suspension was washed via centrifugation (5 min at 1200 rpm) in DMEM/F-12 medium. The cells were then plated at a density of 5 × 10^5^ cells/10 cm standard tissue culture plate in 10 mL of culture medium and cultured in a 5% CO_2_ and 90% humidity incubator at 37 °C for several days until confluence was reached. The medium was changed every 3–4 days. When the cells reached 80% confluence, they were trypsinized and transferred into new 10 cm dishes with a density of 5 × 10^5^ cells/dish. The cells were sub-cultured until passage 1 (P1).

#### 4.2.6. Cell Viability of Rabbit Tenocytes

Specifically, two different types of PRFM (fibrin zone PRFM and platelet-rich zone PRFM) were used to assess their differential effects on cell viability. The P1 tenocytes were suspended in DMEM/F12 medium at a density of 1 × 10^4^ cells/mL per well and loaded on 24-well plate. After 24 h, the medium was removed, and 1 mL of 25%, 50%, or 100% PRFM from either fibrin zone PRFM or platelet-rich zone PRFM were added, respectively. The medium was changed every three days for a total culture period of nine days. To quantify the effects of PRF on cell viability, at each time point (days 3, 6, and 9), the viable cell number in the respective groups was counted using thiazolyl blue tetrazolium bromide (MTT, Sigma-Aldrich, Inc., Carlsbad, CA, USA) following the manufacturer’s instructions. Briefly, MTT reagent was added to each sample and incubated for 3 h to allow the formation of MTT formazan. The resulting formazan was educed with dimethyl sulfoxide (DMSO, Sigma-Aldrich, Inc., St. Louis, MO, USA), and the absorbance of each solution was measured at a wavelength of 595 nm with a microplate reader (Bio-Rad, Hercules, CA, USA) in quadruplicate.

#### 4.2.7. Tenogenic Gene Expression of Cultured Tenocytes

In this experiment, the PRFMs prepared from the platelet-rich zone were used. In addition, the effects of different concentration of PRFM was compared with standard culture medium (DMEM/F-12 containing 10% FBS (10% FBSM)). Briefly, the P1 tenocytes were treated with 10% FBSM, 25%, 50%, or 100% PRFM for a total culture period of six days. Total RNA of cells after various treatments on days 3 and 6 were extracted by the TRIzol^®^ reagent and then stored at −80 °C for later use. RNA (500 ng) was then reverse-transcribed in a 20 μL reaction mixture. Reverse-transcription was performed according to the protocol described by the manufacturer (Superscript III Kit, Invitrogen). Aliquots of cDNA specimens in each group were further amplified by real time PCR for quantitative gene expression levels in a qRT-PCR device (Applied Biosystems) SuperScript III platinum SYBR Green One-Step qRT-PCR kit (Life Technologies, CA, USA). Specific primers for type I collagen (*Col1a1*), type III collagen (*Col3a1*), Tenomodulin (*Tnmd*), Tenascin (*Tnc*), and β-actin are shown in Table 1. The relative change in gene expression was determined via the comparative 2^–ΔΔ^*^C^*^T^ method, where ^Δ^*^C^*^T^ = CT, target - CT, β-actin and ^Δ(Δ^*^C^*^T)^ = Δ*C*T, stimulated ^−Δ^*^C^*^T^, control. The β-actin was used as the internal control, and tendon samples from the control healthy group were used as reference samples.

### 4.3. In Vivo

#### 4.3.1. Surgical Procedure to Create a Tendon Defect

**Figure 7:**
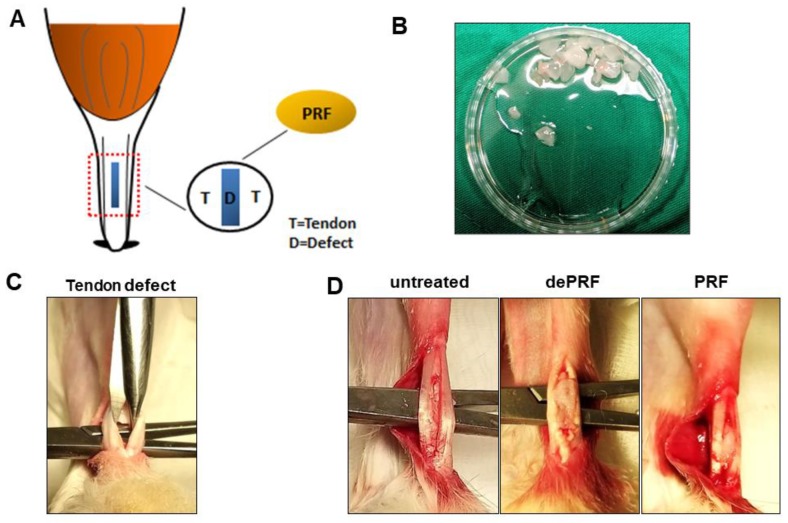
(**A**) Schematic illustration of surgical implantation of platelet-rich fibrin (PRF) into tendon defect. (**B**) Preparation of PRF scaffold. (**C**) The tendon midsubstance is removed with microscissors. (**D**) The created tendon defect was left untreated in control or implanted with heat-denatured PRF (dePRF) or normal PRF scaffold.

The illustration of rabbit Achilles tendon defect healing model was shown in Figure 7A. Eighteen skeletally mature New Zealand White rabbits (male, mean weight 2–2.5 kg) were used. All animals were kept anesthetized with isoflurane in the prone position. A slightly curved 1-cm incision was made lateral to the Achilles tendon to create a skin flap, ensuring that the Achilles tendon would not be injured under the skin wound. Two no. 15 surgical blades (Fisher Scientific, Hampton, NH, USA) bonded with cyanoacrylate were used to make parallel incisions in the tendon 0.5 mm apart and 5 mm long, spanning from the tendon-bone insertion at the calcaneus to the mid-tendon. PRF gel was cut into 0.5 cm^3^ in size before implantation (Figure 7B). Microscissors were used to remove the middle portion of tendon, such that a full-thickness defect was created at mid-substance (Figure 7C). Then, according to group assignment, the defect was left untreated (sham control group), filled with 0.5 cm^3^ of PRF (PRF group) or 0.5 cm^3^ of heat-denatured PRF (dePRF group) (Figure 7D). Finally, the wound was closed with absorbable sutures. Postoperatively, penicillin (40,000 IU/kg for three days) and ketoprofen (2.2 mg/kg for three days) were administered for the prevention of wound infection and pain control in all experimental animals. All rabbits were allowed to move within the cage without restriction after surgery. Rabbits in all three groups were sacrificed under general anesthesia at six weeks post-surgery via intramuscular njection of 10 mg/kg xylazine hydrochloride (BAYER, Leverkusen, Germany) followed by inhalational carbon dioxide. Later, the Achilles tendons were harvested proximally from the musculotendinous junction to the calcaneus distally. For each rabbit, the Achilles tendon from right leg was used for histological analysis, whereas the Achilles tendon from left leg was used for sonographic evaluation.

#### 4.3.2. Sonographic Evaluation

Transverse and longitudinal sonographic images were collected using a 6.7 MHz to 18 MHz linear probe (GE Medical System, L8-18i transducer, Vista, CA, USA). The sonographic echogenicity, homogeneity, and intensity of regenerated tissues were assessed and recorded.

#### 4.3.3. Histological Analysis

For histological evaluation, the tendons were fixed in 10% buffered formalin for three days before routine processing. The samples were then embedded in paraffin and the blocks were cut into serial 5 μm sections followed the long axis of the tendon. The sections were stained with hematoxylin and eosin (H&E) and Masson’s trichrome (MT) (Sigma Aldrich, St. Louis, MO, USA). The tendon healing outcome was compared among each group and evaluated semi-quantitatively using the scoring system described by Nixon et al. [41]. Each parameter was graded on a scale of 1–4, with lower scores correlating to better reparative outcome.

#### 4.3.4. Statistical Analysis

For the quantitative assay, each data point was derived from three independent experiments or an experiment of triplicate assay and was presented as mean with standard deviation. All analyses were performed using GraphPad Prism 8.0 analytic software. Statistical significance was set at a *p* value of <0.05. The qPCR and histological data were analyzed using one-way, two-way ANOVA followed by post hoc scheffe test, multiple comparisons Dunnett test, and Sidak test. The statistical significances among the experimental groups were indicated with asterisks. Groups labeled with asterisk superscript letters indicate that the statistic difference between the two groups had a *p* value of less than 0.05 and was considered significantly different.

## 5. Conclusions

The current study shows that implantation of PRF facilitates the tissue healing in a partial-tendon defect animal model. PRF could serve as a cytokine delivery vehicle in promoting cell viability, proliferation, differentiation and extracellular matrix synthesis. Our data indicate that PRF treatment may accelerate Achilles tendon healing, which would allow for early functional rehabilitation, thereby shortening the recovery period for work or sports activities.

## Figures and Tables

**Table 1:** Primers for rabbit tenogenic marker mRNA detection.

Gene	Primer Sequence	Size (basepair)
*Col1a1* *XM_017348831.1*	F:ACCTGGTCCCCAAGGTTTCCAA R:CTTGGCACCATCCAAACCACTG	247
*Col3a1* *XM_002712333.3*	F:TGGTCTTCCTGGTGAAAACGGA R:TTCACCCTTAGCACCAGGGGAT	196
*Tnmd* *NM_001109818.1*	F:GAACAAAATGAGCAGTGGGTGGTC R:TTGCAAGGCATGATGACACGACAG	272
*Tenascin* *FJ480400.1*	F:AGGGCTTTGAGGAAAGTGAACC R:TCAGAGCATACTCCACCGTGTT	216
*β* *-actin* *NM_001101683.1*	F:CAACTGGGACGACATGGAGAAG R:TGAACGTCTCGAACATGATCTG	152
